# Supplementary material for: Extraction of Polyphenols and Valorization of Fibers from Istrian-Grown Pomegranate (Punica granatum L.)
Source: Foods. 2022 Sep 6;11(18):2740. doi: 10.3390/foods11182740 (PMC9497529; doi:10.3390/foods11182740)
Supplement: Supplementary file 1 [file foods-11-02740-s001.zip › foods-1848983-supplementary.pdf]

Supplementary Figures

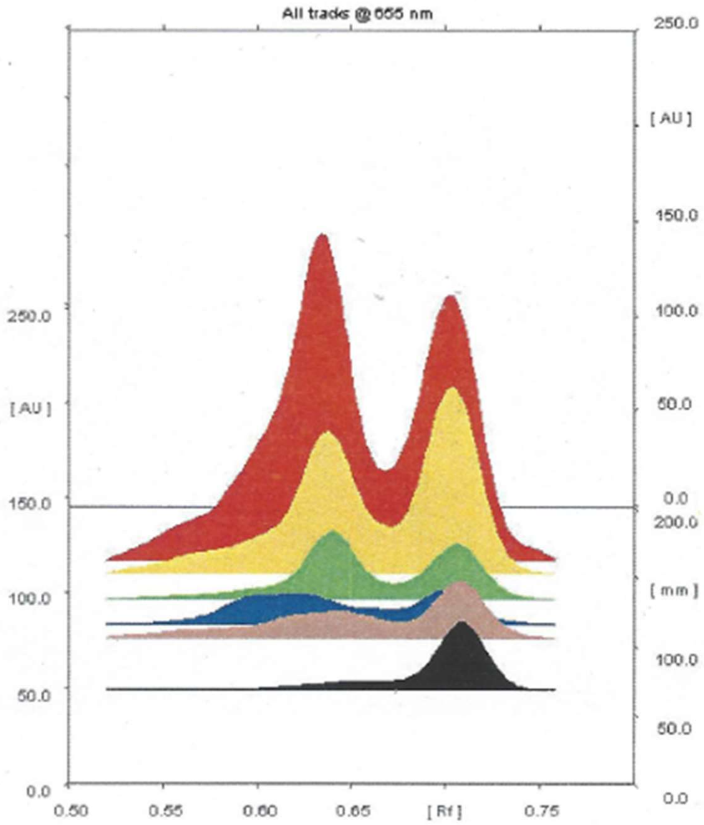

Figure S1. Densitograms of (+) catechin standard (▲) and 70% ethanol pomegranate extracts (▲pomegranate seeds**;** ▲ pomegranate juice; ▲pomegranate membrane; ▲ pomegranate mesocarp; ▲ pomegranate peel) scanned on the HPTLC cellulose plate at 655 nm after derivatization with DMACA reagent. Additionally to described fractions (section 2.2) pomegranate membrane is shown herein as the thin mesocarp layer directly in contact with the arils.

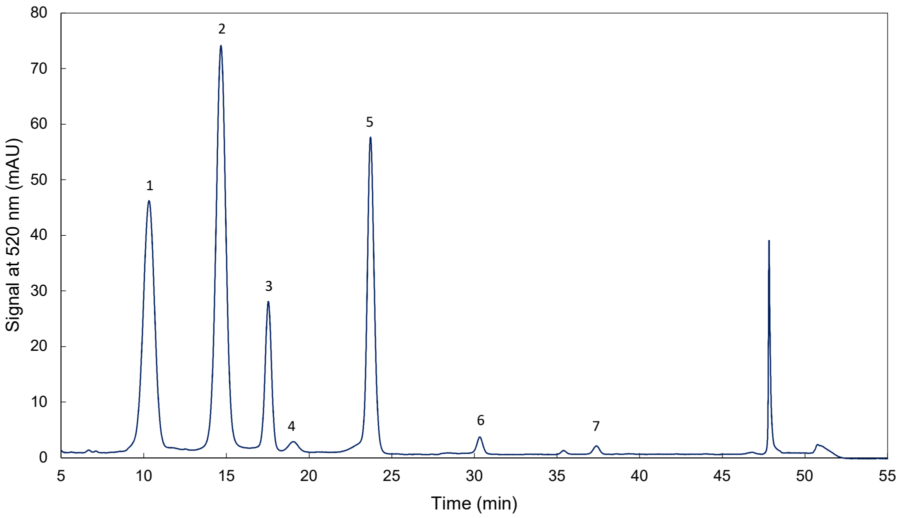

Figure S2. Separation of anthocyanins in pomegranate juice by HPLC. Peeks: (1) Del-3,5-diGly; (2) Cy-3,5-diGly; (3) Del-3-Gly; (4) Pel-3,5-diGly; (5) Cy-3-Gly; (6) Pel-3-Gly, (7) non-determined with HPLC.

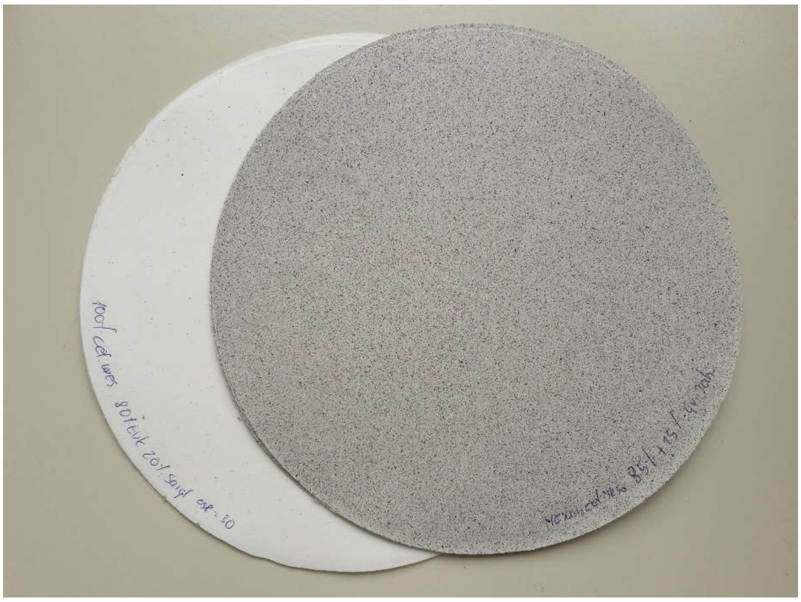

Figure S3. Laboratory samples of paper produced from commercial cellulose fibers (left) and a mixture of cellulose fibers with pomegranate peels as a filler (right).
